# Anthropogenic hybridization between endangered migratory and commercially harvested stationary whitefish taxa (*Coregonus* spp.)

**DOI:** 10.1111/eva.12166

**Published:** 2014-07-08

**Authors:** Jan Dierking, Luke Phelps, Kim Præbel, Gesine Ramm, Enno Prigge, Jost Borcherding, Matthias Brunke, Christophe Eizaguirre

**Affiliations:** 1 Research Division Marine Ecology, Research Unit Evolutionary Ecology of Marine Fishes, GEOMAR Helmholtz Centre for Ocean ResearchKiel, Germany; 2 Department of Evolutionary Ecology, Max Planck Institute for Evolutionary BiologyPlön, Germany; 3 Department of Arctic and Marine Biology, Faculty of Biosciences Fisheries and Economics, University of TromsøTromsø, Norway; 4 Faculty of Science, University of CopenhagenFrederiksberg, Denmark; 5 General Ecology & Limnology, Ecological Research Station Grietherbusch, Zoological Institute of the University of CologneCologne, Germany; 6 Landesamt für Landwirtschaft, Umwelt und ländliche Räume (LLUR)Flintbek, Germany

**Keywords:** admixture, anadromous fish, conservation, evolutionarily significant unit, gill raker, introgression, stocking

## Abstract

Natural hybridization plays a key role in the process of speciation. However, anthropogenic (human induced) hybridization of historically isolated taxa raises conservation issues. Due to weak barriers to gene flow and the presence of endangered taxa, the whitefish species complex is an excellent study system to investigate the consequences of hybridization in conservation. We focused on three naturally reproductively isolated whitefish taxa in Germany: the endangered, anadromous North Sea houting (NSH) and Baltic houting (BH), which were reintroduced after local extinction, and the commercially stocked European whitefish (EW). To evaluate the genetic integrity of each taxon, source and reintroduced populations of NSH and BH, and EW populations were characterized based on two mitochondrial and 17 microsatellite loci. Additionally, we investigated gill raker counts as an adaptive phenotypic trait. Even though clear genetic and phenotypic differentiation confirmed the houtings as separate evolutionarily significant units, admixture analyses revealed an extensive hybrid zone. Hybridizations were introgressive, positively correlated with genetic diversity, and were reflected in the gill raker counts. The BH distribution range showed higher heterogeneity and stronger admixture than the NSH range. Erroneous stocking with non-native genotypes best explained these patterns, which pose challenges for the conservation of the endangered NSH and BH.

## Introduction

The present age has been coined ‘the sixth extinction’ (Leakey and Lewin 1996), as current extinction rates driven by anthropogenic impacts rival those of the five previous mass extinction events on earth. The conservation status of diadromous fishes in Europe is a dramatic example, where one-third are classified as endangered (Kottelat and Freyhof 2007). Cryptic drivers contributing to biodiversity loss are introgressive hybridization and reverse speciation (Seehausen 2006) associated with habitat modifications and species translocations by humans (Allendorf et al. 2001). Particularly, translocation by stocking (i.e., the release of fry reared in hatcheries) can serve to reintroduce endangered species or to increase their population sizes. On the other hand, it can also introduce non-native species or strains that subsequently can hybridize with native taxa and may affect the fitness of wild populations (Consuegra and Garcia de Leaniz 2008; Araki et al. 2009; Hansen et al. 2009).

The role of hybridization in conservation is a dilemma: Whereas natural hybridization plays an important evolutionary role in the process of speciation and the maintenance of biodiversity (Dowling and Secor 1997; Nolte and Tautz 2009), hybridization induced by human activities can be considered as harmful as it threatens the integrity of ancestral species and has contributed directly or indirectly to species extinctions (Rhymer and Simberloff 1996). Allendorf et al. (2001) framed the potential outcomes of such anthropogenic hybridizations into three clearly determined categories: (i) hybridization without introgression (sterile F1s only), (ii) widespread introgression, and (iii) complete admixture, which can result in reverse speciation (Seehausen 2006). Each case poses different conservation challenges, ranging from wasted reproductive effort due to sterile F1s under (i) to the loss of parental lines under (iii) (Allendorf et al. 2001).

The recent postglacial origin of the whitefish species complex (Coregonidae) has resulted in porous barriers to gene flow (Østbye et al. 2005; Præbel et al. 2013a), and natural (Schluter 1996) and anthropogenic hybridizations (Winkler et al. 2011; Bhat et al. 2014) are frequently observed. In Northern Germany, two endangered native anadromous whitefish taxa of considerable conservation interest exist (IUCN 2013): the North Sea houting (NSH) (*Coregonus oxyrinchus* or *Coregonus maraena*, depending on the author) and the Baltic houting (BH) (*Coregonus lavaretus* or *C. maraena*; Freyhof and Schöter 2005; Borcherding et al. 2010). Both taxa are evolutionarily young lineages as they diverged only after the last glaciations, and their species and taxonomic designation is still under debate (Jacobsen et al. 2012). However, Hansen et al. (2008) provided evidence that NSH and BH represent separate evolutionarily significant units (ESUs), that is, groups of organisms to be considered distinct for conservation purposes. Throughout this manuscript, we therefore simply refer to ‘taxa’ and additionally use the term ‘ESU’ in a conservation context. The respective natural ranges of the NSH and BH were the North Sea and Baltic Sea and, during the reproductive period, the rivers in the hydrographically isolated drainage basins of these two seas (Jacobsen et al. 2012). Historically, the houtings sustained fisheries in the Netherlands, Germany, and Denmark (Thienemann 1922; Jennerich and Schulz 2011), but faced extinction by the 1970s due to habitat loss, construction of migration barriers, pollution, and overfishing (Hansen et al. 1999). Since then, conservation programs based on reintroduction by stocking from remnant indigenous source populations have led to the return of the NSH (Jäger 1999; Jepsen et al. 2012) and BH (Jennerich and Schulz 2011) to their historic ranges of distribution. In addition, the Northern German system also includes European whitefish (*C. lavaretus*) (EW), which is commercially stocked in lakes across the same geographic ranges.

The system created by stocking over the past 25 years offers the potential for contemporary secondary contact of three historically geographically isolated whitefish taxa via two possible mechanisms: (i) Anthropogenic translocations outside the historic ranges by erroneous stocking, which could then be followed by diffusion from short-range migrations, termed ‘stepping stone and diffusion’ (Gozlan et al. 2010) and (ii) migrations via the man-made invasion corridor created by the Kiel Canal connecting the North Sea and the Baltic Sea (Gollasch and Rosenthal 2006). The houtings have received recent scientific attention, including studies on evolutionary history (Østbye et al. 2005; Jacobsen et al. 2012), taxonomy (Freyhof and Schöter 2005), conservation genetics in Danish locations (Hansen et al. 2006, 2008), and ecology (Borcherding et al. 2006, 2008, 2014; Jepsen et al. 2012). Yet, the presence of hybridizations between these taxa, and the maintenance of genetic integrity in this system, remains to be investigated. This is surprising considering the porous reproductive barriers within the whitefish species complex. It is also remarkable when considering ongoing large-scale conservation efforts that would benefit from this information, including a €14 million EU LIFE project focused on the NSH in Denmark (Hansen 2006), and large investments in the continuous stocking of German locations.

Here, we assessed the contemporary history of the two endangered houting taxa and the fishery important EW across the German range, based on two mitochondrial (mtDNA) and 17 microsatellite loci. We also assessed gill raker counts (GRCs), which represent a meristic trait related to feeding ecology (Amundsen et al. 2004; Kahilainen and Østbye 2006) that is influenced by diversifying selection (Præbel et al. 2013a) and commonly used in whitefish taxonomy. In light of the controversy regarding the taxonomic and ESU status of houtings, our objectives were to (i) investigate the genotypic and phenotypic integrity of NSH, BH, and EW, (ii) characterize the population genetic structure of this young system, (iii) where present, assess the geographic patterns of hybridizations and their potential underlying anthropogenic drivers (e.g., stocking or the opening of Kiel Canal), and (iv) assess the evolutionary and conservation implications of these findings and provide recommendations for science-based conservation of the endangered NSH and BH in Germany.

## Material and methods

### History of the houting reintroduction

**Figure 1:**
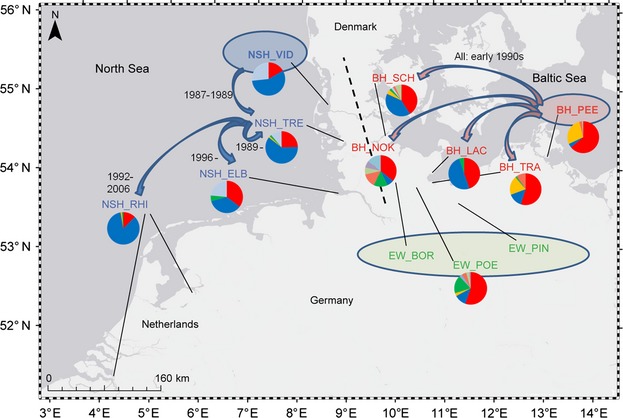
Map of the study area and information on the history of North Sea houting (NSH) and Baltic houting (BH) reintroductions to their former German ranges. Source populations are marked by colored ovals. Arrows depict the official sources and destinations of fry, with the year of the onset and end of stocking. The dotted line represents the watershed separating the historically isolated houting ranges. The pie charts depict the mtDNA haplotype frequencies in each population. Detailed population information is given in Table1. Basemap copyright 2013 Esrl DeLome, NAVTEQ.

The translocation and stocking history as well as the putative sources of fry were obtained for each German NSH and BH population from published (Borcherding et al. 2010) and gray literature (Jäger 1999; Jennerich and Schulz 2011) as well as interviews with hatchery and resource managers. In brief, the NSH was first reintroduced to its historic German distribution range via stocking of fry originating from the indigenous source population in the Danish river Vidå (abbreviated population name including *a priori* taxon affiliation: NSH_VID) to the River Treene (NSH_TRE) in 1987 (Fig.1). Since 1989, adults returning to the Treene River on their spawning migration were used to produce fry to stock the Treene but also the Elbe (NSH_ELB) and Rhine (NSH_RHI) rivers. The BH, on the other hand, was reintroduced by releasing fry originating from the indigenous source population in the River Peene (BH_PEE) in the 1990s to the Schlei (BH_SCH), Trave River (BH_TRA), Kiel Canal (BH_NOK), and Lachsbach (BH_LAC) (Fig.1). Today, the populations BH_PEE, BH_TRA, and BH_NOK are officially stocked with fry from locally caught spawners, whereas BH_LAC and BH_SCH spawners are pooled and resulting fry is released in both rivers. Finally, EW have been stocked in lakes in Northern Germany for commercial fisheries since the 19th century (Thienemann 1922). Supplementary stocking continues in all locations except in the Vidå and the Rhine rivers, where natural reproduction dominates since at least 2005 (Borcherding et al. 2010). The abbreviated population names are summarized in Table1 and will be used throughout this manuscript.

**Table 1:** Sampled populations, sample sizes for different analyses, and basic genetic information from microsatellite analysis.

*A priori* taxon, life history	Population	Abbreviation	Sampling years	Status	Still stocked?	*n* m-sat	*n* mtDNA	*n* gill rakers	*A*_R_	*H*_O_	*H*_E_	*n* HWD	*n* LD
North Sea houting, Anadromous-Marine	Vidå	NSH_VID	2009	Source	No	30	30	–*	5.39	0.62	0.66	2	2
	Treene	NSH_TRE†	2004–2012	Reintroduced	Yes	139	270	86	5.82	0.63	0.67	1	3
	Rhine‡	NSH_RHI	2008	Reintroduced	No	89	72	–	6.58	0.69	0.72	1	2
	Elbe	NSH_ELB	2010	Reintroduced	Yes	16	18	19	7.05	0.63	0.76	5	20
Baltic houting, Anadromous-Brackish	Peene	BH_PEE	2009–2010	Source	Yes	30	97	24	5.74	0.67	0.66	2	1
	Trave	BH_TRA	2009–2011	Reintroduced	Yes	71	62	39	6.61	0.67	0.72	1	3
	Schlei	BH_SCH	2009–2010	Reintroduced	Yes	45	44	19	6.93	0.70	0.74	1	1
	Lachsbach	BH_LAC	2010–2011	Reintroduced	Yes	40	39	5	7.71	0.65	0.74	2	5
	Kiel Canal	BH_NOK	2010–2011	Reintroduced	Yes	10	14	4	–§	0.68	0.67	2	9
European whitefish, Stationary Lake	Lake Bordesholm	EW_BOR	2011	Source?	Yes	30	29	5	6.69	0.68	0.72	0	3
	Lake Pinnow	EW_PIN	2011	Source?	Yes	16	16	–	5.63	0.66	0.70	0	2
	Lake Poenitz	EW_POE	2011	Source?	Yes	26	27	13	6.50	0.75	0.74	2	11
Vendace, Stationary Lake	Lake Selent	vendace	2011	No source¶	Yes	19	–	–					

*n*, sample sizes; *A*_R_, mean allelic richness, rarefied to 28 alleles; *H*_O_ and *H*_E_, mean observed and expected heterozygosity; nHWD, number of loci significantly deviating from Hardy–Weinberg expectations after false discovery rate correction; nLD, number of loci pairs with significant linkage disequilibrium after false discovery rate correction.

* No gill raker counts available; published counts used for comparisons.

† Five time points used for microsatellite analysis, 2004 (*n* = 19), 2009 (*n* = 30), 2010 (*n* = 30), 2011 (*n* = 30), 2012 (*n* = 30).

‡ Three different locations sampled, River Lek (*n* = 30), River Ijssel (*n* = 30), Lake Ijssel (*n* = 29).

§ Excluded from this analysis due to low sample number.

¶ Vendace was not included in further analyses after initial tests showed complete lack of genetic contribution to the houting – EW system.

### Sampling and DNA extraction

We obtained NSH and BH samples from both source populations and all seven reintroduced populations during the winter spawning aggregations in the rivers, and EW samples from three lakes (Table1). Sampling consisted of nonlethal electrofishing and sampling of individuals caught for ongoing stocking programs. For temporal comparisons, NSH_TRE samples from five time points from 2004 to 2012 were collected. Also, to test for substructure in the extensive NSH_RHI system, three small-scale areas were sampled. Vendace (*Coregonus albula*) completed the dataset, as it is the most genetically distinct whitefish taxon in Germany but can occasionally hybridize with EW (Kahilainen et al. 2011). However, after initial tests ruled out hybridization with vendace in our system (Fig. S1), it was excluded from further analyses. Fin clips of all individuals were stored in ethanol, and DNA was extracted using the Qiagen DNeasy kit (Qiagen, Hilden, Germany). A subset of 240 individuals was sacrificed with an overdose of MS-222 to sample gill rakers (Table1). All work was covered by the appropriate permits (fishing: LLUR 31/7174.13.1; sampling: MLUR V 312-7224.121-19).

### Phenotypic analysis

For the distinction and characterization of taxa, GRCs of the first right branchial gill arch were obtained following Kahilainen and Østbye (2006). If this arch was broken, the first left branchial arch was used. Note that left and right GRCs were strongly correlated (Pearson's correlation, *n *=* *31, *r*^2^ = 0.97, *P *<* *0.001). Due to their high protection status, no NSH_VID samples for GRCs were available, and literature GRCs for indigenous NSH from Schöter (2002) were used instead. After verification of normal distribution, differences in GRC between indigenous populations of NSH, BH, and EW were assessed with *t*-tests, and among all populations with an ancova including total length and sex as covariates. Tukey's *post hoc* comparisons were performed. Here and throughout this paper, the false discovery rate was used to correct for multiple tests (Narum 2006) and statistics were conducted using Minitab v.14 (Minitab Inc., Pennsylvania, PA, USA) unless noted otherwise.

### mtDNA analyses

Two mtDNA loci with different mutation rates were used: the NADH dehydrogenase, subunit 3 (ND3, 246 bp) region, and the cytochrome oxidase *b* (cyt *b*, 282 bp). Primers were obtained from Østbye et al. (2005). PCR was performed in 10 *μ*L reactions with 1 *μ*L of template DNA [∼30 ng], 0.5 *μ*L dNTPs (10 mm), 1 *μ*L of each primer (5 mm), 0.1 *μ*L DreamTAQ taq polymerase (Thermo Fischer Scientific Inc., Waltham, MA, USA), 1 *μ*L 10 ×  buffer, and 5.4 *μ*L HPLC water. The PCR cycles started with an initial denaturing phase of 3 min at 95°C, followed by 32 cycles (1 min at 95°C, 45 s at 60°C, 1 min at 72°C), and ended with 10 min at 72°C final extension. PCR products were cleaned with ExoSAP-IT® (Thermo Fischer Scientific Inc.) followed by cycle sequencing using the Big Dye® Terminator v.3.1 kit (Applied Biosystems®, Darmstadt, Germany), and a final purification using the Big Dye® XTerminator™ kit (Applied Biosystems®). Products were sequenced on an ABI 3130 XL Automated Genetic Analyzer (Applied Biosystems®).

Sequences were cleaned and aligned, and both markers were then concatenated into one sequence of 433 bp using CodonCode Aligner v.3.7.1 (CodonCode Corporation, Centerville, MA, USA). DnaSP v.5.10.01 (Librado and Rozas 2009) was used to generate a haplotype data file and to calculate haplotype and nucleotide diversity for each population (Nei 1987). The NETWORK software v.4.6.1.1 (Bandelt et al. 1999) served to create a haplotype network. Population comparisons using Wright's fixation index (*F*_st_) (Wright 1943) based on 1000 permutations were obtained from Arlequin v.3.5.1.2 (Excoffier et al. 2005).

As demographic events can blur evolutionary patterns (Harpending et al. 1998), we tested for recent bottlenecks and population expansions. First, two estimators of neutrality were computed with 1000 coalescent simulations, Tajima's D (Tajima 1989) and Fu's Fs (Fu 1997). Then a sum of squared deviations model of the mismatch distribution was implemented with 100 replicates in a parametric bootstrap approach (Schneider and Excoffier 1999). Finally, the raggedness index r (Harpending 1994) of the observed mismatch distribution was estimated.

### Microsatellite analyses

#### Genotyping and diversity assessment

Samples were genotyped at 17 variable microsatellite loci as detailed in Table S1, which were amplified in three 2.5 *μ*L multiplex PCRs following Præbel et al. (2013b). PCR products were then separated on an ABI 3130 XL Automated Genetic Analyzer. For 120 of 561 samples, the analysis was performed under the same amplification conditions but using 5 *μ*L reaction volumes, and on another sequencer, after running 64 samples on both sequencers for calibration purposes. All alleles were scored by automatic binning in predefined allelic bins followed by visual inspection using GeneMapper v.3.7 (Applied Biosystems). Presence of null alleles and large allele dropout was assessed with Microchecker v.2.2.3 (van Oosterhout et al. 2004).

Deviations from Hardy–Weinberg equilibrium (HWE) and test for linkage disequilibrium were assessed using Arlequin v.3.5.1.2. Finally, the mean allelic richness *A*_R_ (i.e., mean number of alleles per locus) was calculated as measure of genetic diversity harbored by populations using HP-RARE v.1.0 (Kalinowski 2005), and differences in *A*_R_ between source and reintroduced populations were compared with a two-sample *t*-test.

#### Population differentiation

To evaluate the relationship between all NSH, BH, and EW populations, an unrooted neighbor-joining tree using Cavalli-Sforza distances, which are well suited to reveal topologies based on microsatellite frequency data (Takezaki and Nei 1996), was calculated using PHYLIP v.3.69 using 1000 bootstrap replicates (Felsenstein 1989). Then, we evaluated the population structure and pairwise genetic distances among all populations using Wright's *F*_st_ calculated in Arlequin v.3.5.1.2, using 10 000 permutations. Last, an analysis of molecular variance (amova) was run with 1000 permutations to estimate the distribution of genetic variability among *a priori* taxa and populations.

#### Isolation by distance

Under the ‘stepping stone and diffusion’ model of invasion (Gozlan et al. 2010), isolation by distance (IBD) among the reintroduced populations in this study is expected if secondary contact with non-native genotypes is mainly related to migrations. In contrast, contact by stocking alone should not result in IBD (Meraner et al. 2013). We therefore tested for IBD within the historic ranges, using Mantel tests (Mantel 1967) in ibdws v.3.23 with 1000 randomizations (Jensen et al. 2005). Distance matrices were based on Slatkin's (1995) linearized *F*_st_ [*F*_st_/(1–*F*_st_)] from microsatellite data and log-transformed geographic distances between river mouths measured in Google Earth v.7.1.1.1888. Transformations followed considerations in Rousset (1997) on IBD in two-dimensional habitats. We assumed exclusively coastal migrations as proposed by Jepsen et al. (2012).

#### Bayesian population structure

To overcome the possible bias of *a priori* grouping of individuals into taxa or differentiated populations, we used the Bayesian approach implemented in STRUCTURE v.2.3.4 (Pritchard et al. 2000), using an admixture model assuming correlated allele frequencies. We varied the number of possible clusters *K* represented by the sampled individuals from 1 to 6 and ran five independent MCMC simulations to ensure the consistency of results, with a burn-in of 50 000 followed by 100 000 iterations for each *K*. All runs were consistent, and run lengths were sufficient for convergence in all cases, as indicated by STRUCTURE summary statistics (data not shown). The most likely *K* was determined based on Δ*K* (Evanno et al. 2005) and the estimated probability of the data P(D) (Falush et al. 2003), retrieved from STRUCTURE Harvester v.0.6.93 (Earl and vonHoldt 2012). Initial tests including vendace used a subset of 13 microsatellite loci, due to limited cross-amplification of loci among these taxa (Præbel et al. 2013b). As vendace clearly clustered separately from the NSH, BH, and EW (Fig. S1), the subsequent analyses omitted this taxon.

#### Detection and characterization of hybridizations

Hybridizations among the three different taxa in this study (NSH, BH, and EW) were assessed following Latch et al. (2011) with modifications to reflect the presence of three sources. Specifically, five replicate STRUCTURE runs for the most likely number of clusters were used to calculate the mean contributions of NSH (*q)*, BH (*r*), and EW (*s*) and their 90% credible intervals (CIs) to each individual's genotype. Two methods were then used to assign individuals as pure or hybrids. First, under ‘STRUCTURE relaxed’, based on a hybrid threshold of 0.10, individuals were assigned as pure NSH, BH, or EW if either *q*, *r,* or *s *≥* *0.90, and as hybrids showing genotypic contributions of two different taxa if *q, r, and s *<* *0.90 and either *q* and *r*, *q* and *s*, *or r* and *q *>* *0.10. In addition, individuals showing genotypic contributions of all three taxa in this study (i.e., 0.10 <  *q*, *r*, *s *<* *0.90) were defined as compound hybrids. With the number of microsatellite loci and the observed *F*_st_s in our study, the applied thresholds should allow for efficient hybrid identification according to Vähä and Primmer (2006). Second, ‘STRUCTURE conservative’ was calculated as highly conservative method likely to underestimate hybrid proportions but avoiding misclassifications as hybrids (Latch et al. 2011). Here, individuals were designated as pure if one of the CIs around *q*, *r* and *s*- included 1, and all others as hybrids comprising genotypic contributions of at least two taxa. Linkage disequilibrium, which is expected if hybrids are mainly F1s and if hybridization is not introgressive (Latch et al. 2011), was used to confirm introgression.

The extent of admixture was compared between the NSH and BH distribution ranges based on (i) the proportions of pure native, pure non-native and hybrid individuals using a chi-square test, (ii) the mean contribution of the native genotype (NSH range: *q*; BH range: *r*), and (iii) the mean admixture proportion (1 – the proportion of the dominant genotype *q*, *r,* or *s*) in each of the two ranges with two-sample *t*-tests. The latter two parameters were then also compared among reintroduced populations within the distribution ranges, among the years 2004–2012 for the NSH_TRE, and among the three NSH_RHI sites with anovas and Tukey's *post hoc* tests.

#### Underlying mechanisms of geographic patterns in admixture

The two alternative explanations for secondary contact in the study system were migrations via Kiel Canal and translocations by erroneous stocking. To distinguish between them, we assessed the genotypes present in the canal population BH_NOK, with the expectation of a mixture of NSH and BH genotypes if the canal serves as migration corridor. We also analyzed the correlation of the mean proportion of non-native genotype (NSH range: *r *+ *s*; BH range: *q *+ *s*) and of non-native houting genotype (NSH range: 1 – *r*; BH range: 1 – *q*) within populations with distance from the canal, with the expectation of negative correlations if the canal serves as corridor.

#### Hybridization, genetic diversity, and GRCs

To determine whether and how admixture affects genetic diversity, we ran a linear regression analysis between the mean allelic richness *A*_R_ and the mean admixture proportion of populations. Finally, to assess whether hybridizations were reflected in phenotypic traits, we analyzed the correlation of the mean EW genotype proportion with the mean GRC of populations. Initially, all five BH and the two EW populations for which GRC were available were included in the analysis, whereas two available NSH populations were excluded to avoid a confounding influence of differences in GRC between NSH and BH. To test the robustness of results, we then successively excluded EW populations and the strongly admixed BH_NOK population from the analysis. Higher GRCs are expected in hybrids with EW contribution due to the elevated GRCs of pure EW.

## Results

### Phenotypic differentiation based on GRC

**Figure 2:**
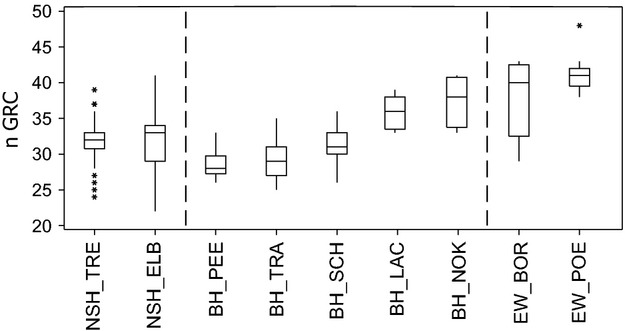
Boxplots of gill raker count (GRC) distributions in reintroduced populations across the North Sea houting (NSH) and Baltic houting (BH) ranges, and in the BH_PEE and European whitefish (EW) source populations. For the source NSH_VID, no gill rakers were available.

When comparing the source populations, the indigenous BH population BH_PEE was characterized by slightly but significantly lower GRC than indigenous NSH populations (Table S2; *T*_46_ = 2.16, *P *=* *0.036). Secondly, GRC of indigenous BH and NSH populations were both significantly lower than EW GRC (BH: Table2; NSH versus EW_BOR: *T*_4_ = −3.05, *P *=* *0.038; NSH versus EW_POE: *T*_22_ = −14.95, *P *<* *0.001). The situation among reintroduced populations differed between the distribution ranges (Fig.2). For NSH, the GRCs in the NSH_TRE and NSH_ELB were comparable with literature values for indigenous NSH, albeit with wider trait variation (Table2 and S2). In contrast, strong interpopulation differences characterized the BH distribution range. While GRCs in the BH_TRA were comparable with those in its putative BH_PEE source (Tukey's *post hoc* test; *t* = 0.56, *P *=* *0.99), the BH_SCH was comparable with the NSH populations NSH_TRE (*t* = −1.07, *P *=* *0.99) and NSH_ELB (*t* = −0.81, *P *=* *0.99), and the BH_NOK as well as BH_LAC with the EW population EW_BOR (all pairwise comparisons summarized in Table2). Fish size (ancova, *F*_1,243_ = 1.63, *P *=* *0.204) and sex (*F*_2, 243_ = 1.41, *P *=* *0.220) were not associated with GRC.

**Table 2:**
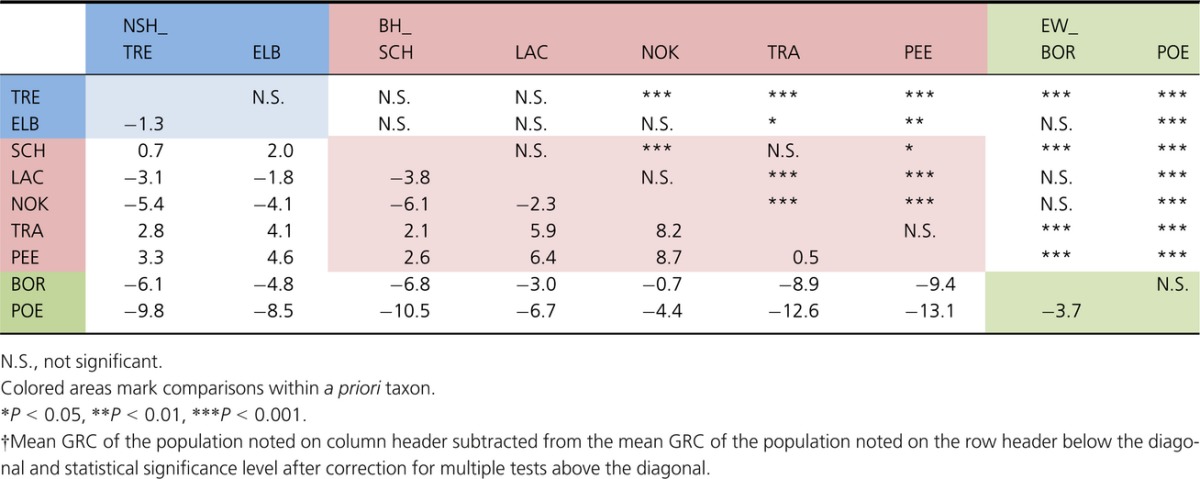
Pairwise differences between source and reintroduced populations of North Sea houting (NSH), Baltic houting (BH), and European whitefish (EW) based on gill raker counts (GRC)†.

### Differentiation and demographic events based on mtDNA

The 723 samples grouped into 21 haplotypes, 10 of which were singletons (six in EW, two in NSH and BH). The remaining 11 haplotypes were dominated by two haplotypes differentiated by seven-point mutations with a combined coverage of 78.4% (Hap 1 – 36.18% and Hap 2 – 42.23%) over the dataset (Fig. S2). On average, Hap 1 was more common in *a priori* BH (55.3%) than NSH (22.1%) and EW (43.9%), and Hap 2 more dominant in *a priori* NSH (63.6%) than BH (18.5%) and EW (14.0%). Mapping haplotypes revealed a gradient across the reintroduced populations (Fig.1).

*F*_st_ values proved to be high and significant between the sources NSH_VID and BH_PEE (*F*_st_ = 0.72, *P *<* *0.001) and between both of these sources and EW (NSH_VID and EW_BOR, *F*_st_ = 0.45, *P *<* *0.001; BH_PEE and EW, *F*_st_ = 0.145, *P *<* *0.001). Except between the NSH_RHI and NSH ELB (*F*_st_ = 0.146, *P *<* *0.001), within the NSH distribution, no significant differentiation among reintroduced populations could be observed. This was not the case within the BH range where the BH_TRA was the only reintroduced population not significantly differentiated from the putative source population BH_PEE (*F*_st_ = 0.03, *P *=* *0.036, versus *F*_st_ > 0.327, *P *<* *0.001 for all other BH populations). We also found that BH_SCH, BH_LAC, and BH_NOK were strongly differentiated from their putative source BH_PEE, but not from NSH or EW populations, respectively (all pairwise comparisons summarized in Table S3). Investigating possible demographic events failed to detect any consistent sign of recent bottlenecks or expansions in any of the populations (Table S4).

### Differentiation and population structure based on microsatellites

**Figure 3:**
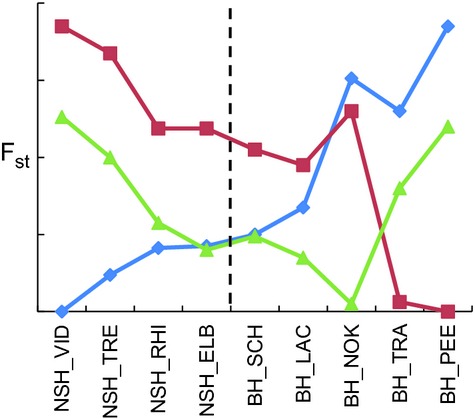
Genetic differentiation (*F*_st_ – values based on microsatellites) of source populations from reintroduced populations across the historic North Sea houting (NSH) (left of dotted line) and Baltic houting (BH) (right of dotted line) ranges. Putative source of NSH reintroductions: NSH_VID (blue); putative source of BH reintroductions: BH_PEE (red); European whitefish (EW) from EW_BOR (blue) is added as potential additional source.

**Figure 4:**
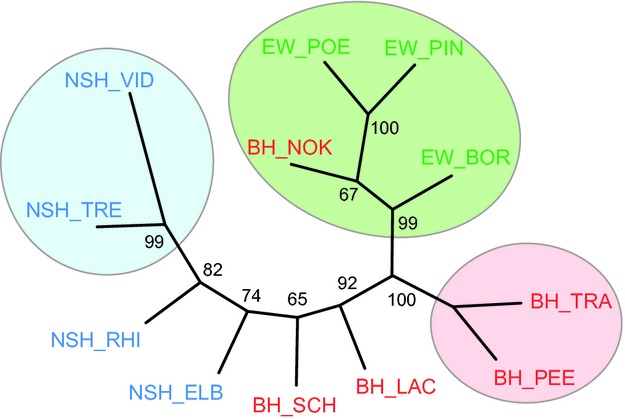
Phylogenetic tree of all source and reintroduced populations of North Sea houting (NSH), Baltic houting (BH), and European whitefish (EW) based on Cavalli-Sforza distances calculated from the microsatellite dataset; shaded circles indicate sources and closely related populations. Node support values represent percentages of 1000 bootstrap replicates across all loci.

While some loci showed deviations from HWE for individual populations, no general patterns were present and all loci were included in the analyses. The genetic integrity of the indigenous source populations detected with mtDNA also held true when investigating nuclear loci (Table3). Specifically, the sources of NSH and BH and EW populations were strongly and significantly differentiated from each other (NSH_VID versus BH PEE, *F*_st_ = 0.15, *P *<* *0.001; *F*_st_s > 0.10 and *P *<* *0.001 for all pairwise comparisons of NSH_VID and BH_PEE versus EW populations) (Table3). Contemporary gene flow among populations within distribution ranges proved to be very low with all pairwise population tests being significant except NSH_ELB versus NSH_RHI (*F*_st_ = 0.01, *P *=* *0.30), BH_SCH versus BH_LAC (*F*_st_ = 0.01, *P *=* *0.06) and BH_TRA versus BH_PEE (*F*_st_ = 0.01, *P *=* *0.054). Interestingly, the latter comparison is the only one showing no significant differentiation from the source population within a given distribution range, and all reintroduced populations showed intermediate differentiation between the putative source and other source populations (Fig.3). The differences between the historic distribution ranges and among populations within ranges both explained significant and approximately equal proportions of molecular variance (amova, Table S5). The general pattern of population clustering within the distribution ranges of NSH and BH was confirmed by the neighbor-joining tree (Fig.4). Interestingly, both *F*_st_ values and the neighbor-joining tree showed that the population from the Kiel Canal, contrary to expectation, clearly clustered with EW and not within the BH range.

**Table 3:**
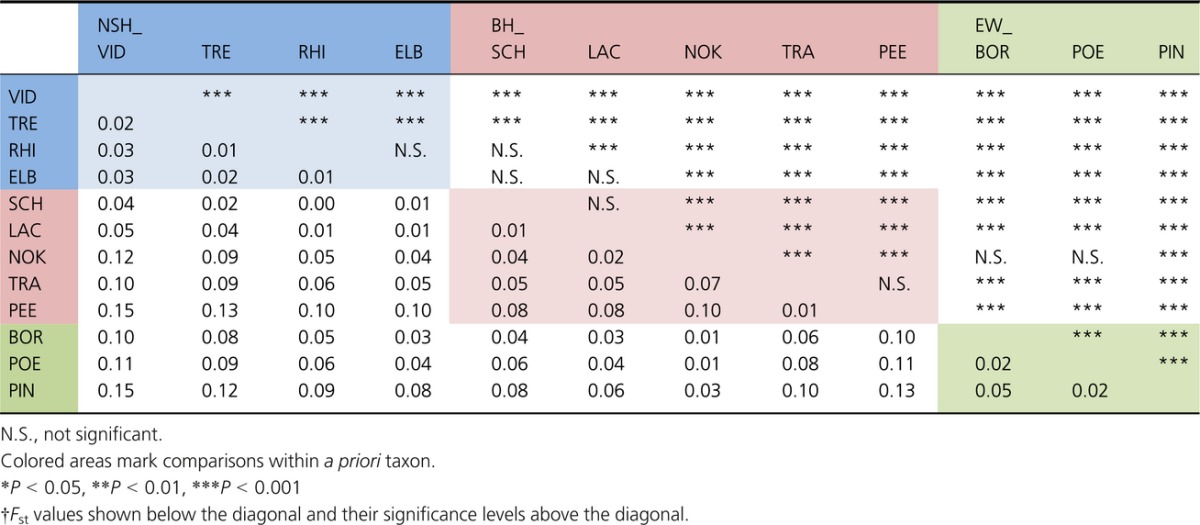
Estimates of pairwise genetic differentiation (*F*_st_) between source and reintroduced populations of North Sea houting (NSH), Baltic houting (BH), and European whitefish (EW) based on microsatellites†.

**Figure 5:**
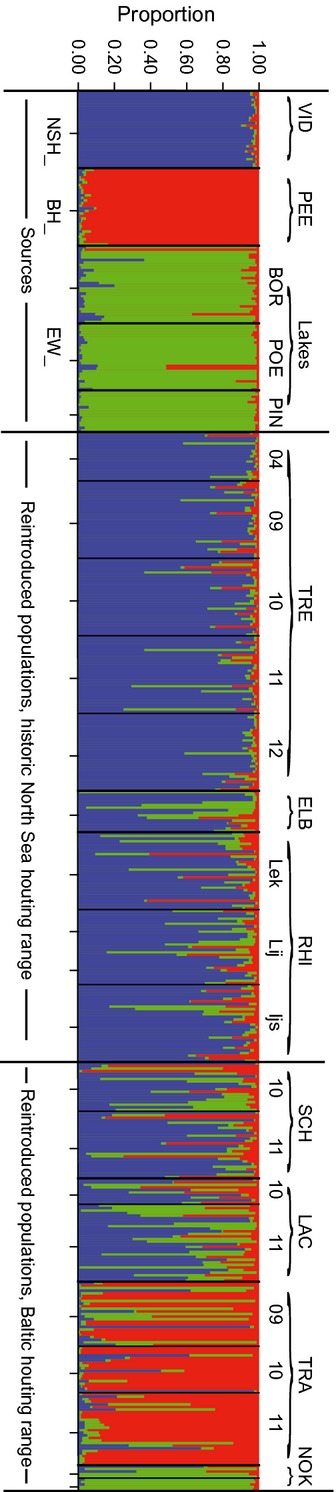
Bayesian clustering of all individuals obtained with STRUCTURE 2.3, assuming the presence of three clusters (*K* = 3). Each vertical bar represents one individual, and the three colors denote the inferred proportional genotypic contributions of each cluster. Potential source populations for reintroductions to the historic ranges are put on the left and all reintroduced populations on the right side of the figure. According to official information, NSH_VID was used for reintroductions to the historic NSH range and BH_PEE to the historic BH range.

Overall, the STRUCTURE analysis revealed *K *=* *3 as most likely number of clusters represented by the sampled individuals after the exclusion of vendace (Fig. S3). The analysis confirmed the dominance of native NSH genotypes across the historic NSH range, but also revealed the additional influence of both EW and BH genotypes in reintroduced populations (Fig.5). No temporal differences in the contribution of NSH genotype in the NSH_TRE were apparent over the period 2004–2012 (anova; *F*_4,138_ = 0.78, *P *=* *0.538), suggesting that admixture occurred before 2004 or elsewhere than in the Treene River. Within the NSH_RHI system, no spatial structuring was detected (*F*_2,88_ = 0.34, *P *=* *0.711).

STRUCTURE unravelled a more complex and heterogeneous situation across the historic BH range of distribution (Fig.5): While the BH_TRA reflected its putative BH_PEE source, the BH_SCH and BH_LAC showed a strong NSH genetic contribution and the BH_NOK a strong EW genetic contribution. Taken all together, results call for in-depth analyses of sources and causes of admixture.

### Presence and characterization of hybridizations

**Figure 6:**
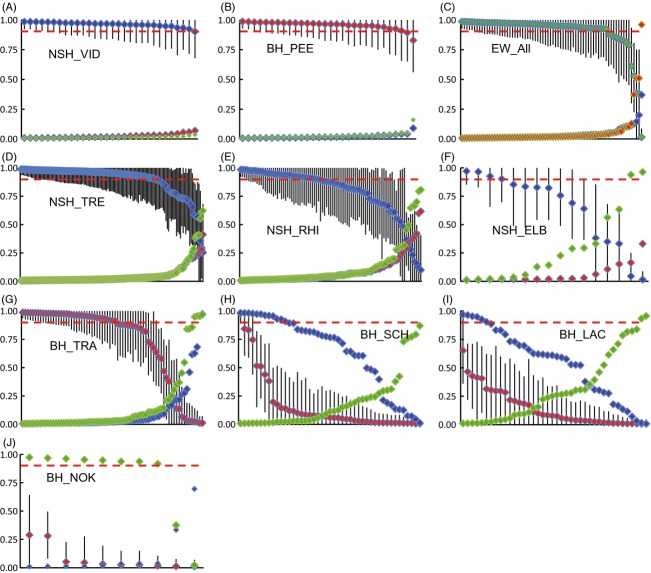
Mean North Sea houting (NSH) (blue), Baltic houting (BH) (red), and European whitefish (EW) (green) proportional contributions to each individual's genotype in each population, based on individual admixture analysis in STRUCTURE 2.3 with the number of clusters fixed to *K* = 3. The putative source populations NSH_VID and BH_PEE and potential additional source EW are displayed at the top for reference, reintroduced populations below. 90% credible intervals (CIs) are given for the *a priori* native genotype at each location. Individuals with a NSH, BH, or EW contribution >0.9 (marked by dotted red lines) were considered pure for that genotype, all others as hybrids, using the STRUCTURE-relaxed threshold. Proportions of the native genotype were ranked along the *x*-axis from highest to lowest.

**Figure 7:**
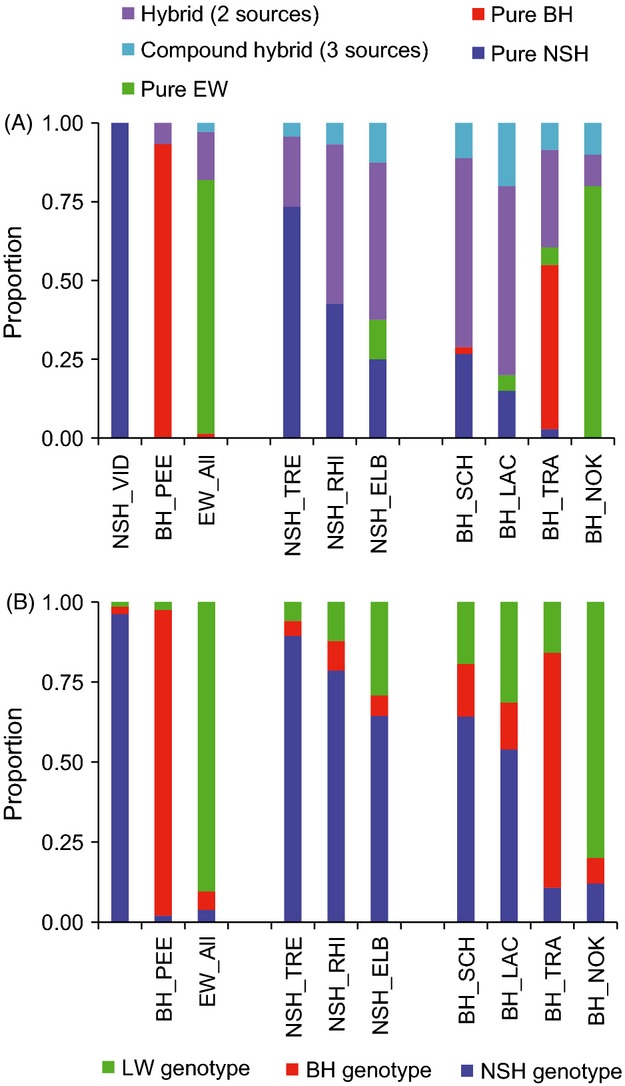
Genetic composition of source populations (left block) and reintroduced North Sea houting (center) and Baltic houting (BH) populations based on analysis in STRUCTURE 2.3, assuming *K* = 3 clusters. (A) Proportion of pure and hybrid individuals in each population identified based on STRUCTURE-relaxed thresholds. ‘Compound hybrids’ denote individuals with genetic contributions of all three sources. (B) Mean contribution of NSH, BH, and EW genotype to each population.

Individual admixture analysis in STRUCTURE (Fig.6) revealed a previously unrecognized hybrid zone involving all three taxa NSH, BH, and EW (Fig.7). The source populations NSH_VID and BH_ PEE, and with some exceptions EW, consisted of pure individuals of the *a priori* expected genotype (100%, 93–100%, and 80–93%, respectively) (Fig.7, Table4). In contrast, all reintroduced populations were admixed and harbored compound hybrids (Fig.7, Table4), but with a significantly lower proportion of admixture (*t*-test; *t*_222_ = −13.15, *P *<* *0.001) and of pure non-native individuals (chi-square test; 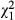 = 73.56, *P *<* *0.001) and hybrids (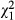 = 32.26, *P *<* *0.001) in the NSH distribution range compared with the BH range. Within the distribution ranges, for NSH, admixture proportions differed significantly and ranged from 11% in the NSH_TRE to 36% in the NSH_ELB (anova; *F*_2,243_ = 18.14, *P *<* *0.001). For the BH, the admixture proportion was 27% in the BH_TRA, but significantly higher (85% and 84%, respectively) in the BH_LAC and BH_SCH (anova; *F*_2,165_ = 62.85, *P *<* *0.001). In addition, hybrids and non-native genotypes dominated in the BH_LAC and BH_SCH, and non-native EW genotype in the BH_NOK population (Figs6 and 7).

**Table 4:** Pure and hybrid individuals in source and reintroduced populations identified with STRUCTURE 2.3, based on STRUCTURE-relaxed (R) and STRUCTURE-conservative (C) thresholds*.

	pure NSH		pure BH		pure EW		Hybrid		Total
	R	C	R	C	R	C	R	C	R, C
Sources									
NSH_VID	30	30	0	0	0	0	0/0	0	30
BH_PEE	0	0	28	30	0	0	2/0	0	30
EW_All	0	0	1	1	58	67	13/2	4	72
NSH reintroduced									
NSH_TRE	102	116	0	0	0	1	37/6	22	139
NSH_RHI	38	61	0	0	0	3	51/6	25	89
NSH_ELB	4	8	0	0	2	2	10/2	6	16
NSH_all	144	185	0	0	2	6	98/14	53	244
BH reintroduced									
BH_TRA	2	2	37	48	4	7	28/6	14	71
BH_LAC	6	10	0	0	2	7	32/8	23	40
BH_SCH	12	23	1	3	0	4	32/5	15	45
BH_NOK	0	0	0	0	8	8	2/1	2	10
BH_all	20	35	38	51	14	26	94/20	54	166
Total	194	250	67	82	74	99	207/36	111	542

* Thresholds under both approaches are described in detail in the Materials and Methods section. Generally, based on genetic differentiation and number of markers in this study, STRUCTURE relaxed is expected to offer good accuracy and high efficiency of hybrid identification, whereas STRUCTURE conservative offers very good accuracy but low efficiency (i.e., it will not classify individuals falsely as hybrids but underestimates the number of hybrids). For R, the hybrid category includes the total number of identified hybrids (left) and the number of hybrids representing compound hybrids with genetic contributions of all three sources (right). The latter was not determined in C.

The number of alleles in linkage disequilibrium in the reintroduced populations of both NSH and BH was comparable with that in the pure source populations, with the exception of elevated numbers in the NSH_ELB, BH_LAC, and BH_NOK (Table1).

### Underlying mechanisms of geographic patterns in admixture

The proportions of non-native genotype (Pearson's *r *=* *−0.28, *P *=* *0.585) and non-native houting genotype (Pearson's *r *=* *−0.19, *P *=* *0.71) in reintroduced populations across the NSH and BH distribution ranges were not correlated with geographic distance from the canal. Furthermore, the most predominant genotype in the Kiel Canal, contrary to expectation, was the EW genotype and not a mix of NSH and BH (Fig.7). Within both the NSH (Mantel test; *Z* = 0.09, *r *=* *−0.99, *P *=* *1.00) and BH (*Z* = 0.50, *r *=* *−0.09, *P *=* *0.622) distribution ranges, IBD between reintroduced populations could not explain the observed genetic structuring.

### Hybridization, genetic diversity, and GRCs

**Figure 8:**
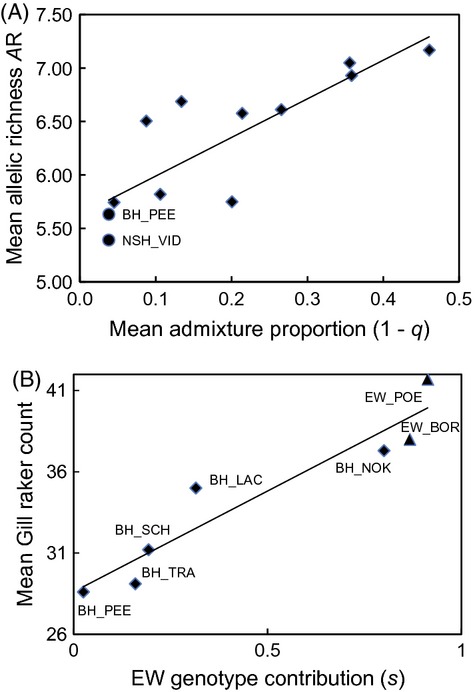
(A) Mean allelic richness *A*_R_ plotted versus the mean admixture proportion (1 – the dominant genotype at a location, i.e., 1 – *q*, *r* or *s*) of populations. Source populations of North Sea houting (NSH) and Baltic houting (BH) are displayed as circle symbols. Regression line *r*^2^ = 0.69. (B) Mean gill raker counts plotted against the mean genetic contribution of European whitefish (EW) to populations, with all available BH (diamond symbols) and EW (triangles) populations included. Population labels are added in the figure. Regression line *r*^2^ = 0.89.

The mean allelic richness *A*_R_ in reintroduced populations clearly surpassed the sources NSH_VID and BH_PEE (mean of 6.69 vs 5.57; *t*_3_ = −4.25, *P *<* *0.024) (Table1). The pattern was largely driven by differences in admixture proportion (Fig.8A; regression analysis, *F*_1,11_ = 22.64, *P *<* *0.001, *r*^2^ = 0.69).

Furthermore, the mean GRC and the mean proportion of EW genotype in a population were strongly correlated independent of whether all BH and EW populations (Pearson's *r *=* *0.947, df = 6, *P *=* *0.001), only the BH populations (*r *=* *0.911, df = 4, *P *=* *0.031), or only BH without the highly admixed BH_NOK population (*r *=* *0.916, df = 3, *P *=* *0.084) were included in the analysis (Fig.8B).

## Discussion

### Differentiation of North Sea houting, Baltic houting, and European whitefish

Debate regarding the species and evolutionary significant unit status has surrounded the houtings (Freyhof and Schöter 2005; Jepsen et al. 2012). Here, the distinctness of the three sources NSH_VID, BH_ PEE, and EW was sustained by multiple lines of evidence: (i) phenotypic differences in GRC, which is often used as a taxonomic trait, (ii) strong and significant genetic differentiation between the indigenous source populations based on both mtDNA and microsatellites, (iii) identification of *K *=* *3 clusters with no *a priori* assumption on the population of origin, and (iv) the homogenous structure of the source populations (with some exceptions for EW) revealed by STRUCTURE. Our results thus clearly support that for the ancestral/source populations of NSH and BH, the conservation classification as separate ESUs (Hansen et al. 2008) holds independently of the actual species status.

### Presence and characterization of hybridizations

Even though the genetic integrity of the source populations of NSH, BH, and EW was maintained, our study documented the widespread presence of three-way hybridizations. Hybridization was shown by the intermediate genetic differentiation of admixed populations from the sources and the increased genetic diversity in identified admixed populations. This discovery was surprising, considering the natural geographic isolation of these taxa (Jacobsen et al. 2012), their homing behavior, and the generally well-described hybrid zones in the whitefish complex (Bernatchez and Dodson 1994; Rogers et al. 2007; Kahilainen et al. 2011; Vonlanthen et al. 2012). Individual admixture analysis showed that all reintroduced German populations of NSH and BH carry some level of admixture, even when applying conservative thresholds. Genetic drift is an unlikely alternative explanation, as it should result in decreased genetic diversity in reintroduced populations and in increased divergence from the putative source, not necessarily in the direction of alternative sources.

Hybrids were viable and hybridizations thus introgressive following Allendorf et al. (2001). This was demonstrated by (i) the frequent occurrence of compound hybrids comprising genotypic contributions of three different taxa, that is, a signature that requires at least two generations of hybridization, and (ii) the presence of pure NSH and hybrids but lack of pure BH or EW, for example, in the NSH_RHI and NSH_TRE, which pointed to past introgression as both parental lines would have to be present if hybridization is ongoing. Further confirmation came from the similar number of linkage and Hardy–Weinberg disequilibria in most reintroduced populations compared with sources, inconsistent with the presence of mainly F1 hybrids or very recent admixture (Barton and Hewitt 1985). Hybridization of NSH with EW was previously shown to the F1 stage (Hansen et al. 2008). Here, we extended those findings by showing the introgressive nature and hybridizations between all three taxa across the complete German distribution area.

### Underlying causes of geographic patterns in admixture

Considering their historic geographic isolation (Jacobsen et al. 2012), what brings NSH, BH, and EW into secondary contact? The system here was broadly characterized by (i) the lack of admixture in the NSH and BH source populations and – with few exceptions – in the EW populations, but the presence of admixture in all reintroduced populations; (ii) the significant differences between reintroduced NSH and BH populations, with the dominance of the *a priori* native genotype and relatively homogenous patterns across the NSH range, and the contrasting heterogeneous situation and ‘out-of-place’ populations across the BH range.

Pattern (i) confirmed that hybridizations were not due to historic admixture in the source populations, but must have occurred after the onset of reintroductions to Germany ∼20–25 years ago. The two possible explanations were anthropogenic translocations via erroneous stocking, or migrations via the potential invasion corridor Kiel Canal (Gollasch and Rosenthal 2006). However, the observed geographic patterns and spatial analyses (patterns i and ii) clearly implicated stocking as key mechanism. Indeed, if the Kiel Canal had created any link for migrations of houtings between the North Sea and the Baltic Sea basins, the local population would not be dominated by the EW genotypes, and non-native genotypes would have been more prevalent in populations near the canal. Moreover, the lack of IBD and of a ‘stepping stone and diffusion’ pattern (Gozlan et al. 2010) within the distribution ranges indicated very limited migrations even between neighboring populations. This was in line with an extensive Danish NSH tagging program reporting few recaptures outside the native river (Jepsen et al. 2012). These findings underscore the idea that the homing behavior of the houtings is strong.

Interviews with resource managers revealed that adults, fertilized eggs, and fry of NSH, BH, and EW have been periodically kept in the same facilities. Although official records did not indicate stocking of the historic houting ranges with non-native ESUs, stocking errors therefore appear possible. The clear range-specific differences observed here may relate to the fact that the same person has been responsible for NSH reintroductions from the onset (Jäger 1999), whereas several hatcheries have been responsible for stocking of the BH range. The potential consequences of too many hatcheries involved are illustrated by the weak differentiation of the BH_LAC from the distant BH_SCH population, both of which are stocked by the same hatchery, but stronger differentiation from the nearby BH_TRA population stocked by a different hatchery.

The intensity of stocking with non-native genotypes strongly influences the outcome of hybridizations (Salminen et al. 2012), but pre- or postzygotic isolating mechanisms also commonly play a role (Hansen et al. 2009; Winkler et al. 2011). The rapid spread of extensive introgressive hybridization within <25 years – that is, few generations considering the maximum age for NSH of 12 years and first reproduction at 2–4 years (Jepsen et al. 2012) – was inconsistent with the presence of strong hybrid incompatibilities *sensu* Orr (1995). Yet, the persistence and sometimes dominance of pure native genotypes and the rarity of hybrid swarms may point to mechanisms opposing introgression. A combination of stocking program and river system characteristics may thus ultimately determine the outcome of hybridizations; however, deeper ecological characterization is needed to assess the respective contributions conclusively.

### Evolutionary and conservation implications

From the evolutionary perspective, following Hewitt (1988), the newly described NSH–BH–EW hybrid zone represents in essence a large natural experiment. The potential fitness implications of hybridizations were demonstrated by the strong correlation of GRC with admixture proportions, as GRCs directly relate to feeding ecology in whitefish (Amundsen et al. 2004; Kahilainen and Østbye 2006) and are therefore under selection (Præbel et al. 2013a). Hybrids may then perform differently and could even potentially exploit different niches, compared with pure individuals in the same system. Moreover, this system is special compared with many other hybrid zones including those involving whitefishes (Winkler et al. 2011; Meraner et al. 2013), because the three genetically and phenotypically different taxa and their hybrids now occur sympatrically in three very different environments: the fully marine North Sea, the brackish Baltic Sea, and freshwater rivers. Each environment will exert divergent selection pressures notably on immune systems (Eizaguirre et al. 2009) and osmoregulation (Ban et al. 2007; Papakostas et al. 2012). This setup appears ideal to investigate questions regarding the role of environment-dependent selection against or for hybrids and disruption of local adaptations in shaping hybrid zones (Hendry et al. 2000; Nolte et al. 2006).

In contrast to declines in genetic diversity frequently associated with stocking due to bottlenecks (Pister 2001), diversity actually increased in stocked compared with indigenous populations due to admixture, similar to the situation in managed honeybees (Harpur et al. 2012). Even though anthropogenic hybridizations are in most cases detrimental, this raises the question whether admixed populations may possess an enhanced evolutionary potential (Mallet 2007). Whether the fate of NSH and BH system will be persistence of locally adapted ESUs, homogenous hybrid swarms, or divergence into new niches is a key question (Nolte and Tautz 2009). The answer will at least partly depend on the course of future conservation measures, which should particularly consider the gene flow imposed on local populations by introducing novel genetic material by stocking for instance (Aitken and Whitlock 2013).

The confirmation of the previous classification of NSH and BH as separate ESUs (Hansen et al. 2008) underscores that separate management of the two groups should be maintained. This conclusion stands independently of the low reproductive isolation between NSH, BH, and EW, which is in accordance with the generally weak barriers to gene flow among whitefishes (Vonlanthen et al. 2012). The newly described hybrid zone will complicate this task and presents a significant conservation challenge. Based on our findings, we provide several concrete resource management recommendations to assist the future conservation of the houtings: (i) Hybridizations in the endangered houtings likely stemmed from erroneous stocking, which demonstrates the need for a stronger conservation genetic monitoring of stocking programs and hatcheries; (ii) the overall continued genetic integrity of the NSH and BH source populations should be a goal to maintain ESUs, which is a conservation priority in hybrid zones (Allendorf et al. 2001). Any stocking of source locations with fry from reintroduced populations thus needs to be prevented to protect these pure native populations; (iii) pure genotypes may also persist in the reintroduced populations, as evidenced by the continued dominance of native genotype in all NSH populations and the BH_TRA, and the temporally stable situation in the NSH_TRE. Although reverting admixed populations to exclusively pure genotypes may not be feasible, using spawners with pure native genotypes for stocking programs could further decrease admixture proportions (Hansen et al. 2009). In the long term, shifting conservation priorities to habitat restoration may contribute to this end, as it could strengthen the role of natural selection for locally adapted ESUs. Finally, the continued temporal genetic monitoring of this system in the future will be imperative to assess the success of houting conservation programs.

## Data archiving statement

Raw data for this study are available from the Dryad Digital Repository: http://dx.doi.org/10.5061/dryad.n62 dm

## Supporting Information

Figure S1 Bayesian clustering of individuals with vendace (V) included in the dataset.

Figure S2 Haplotype network based on combined ND3/cyt *b* mtDNA sequences (433 bp).

Figure S3 Probability of the number of clusters represented by the microsatellite dataset based on five replicate runs in STRUCTURE 2.3, as indicated by the *ad hoc* statistic Δ*K* (gray datapoints) and Ln P(D) (mean ± 1 SD, black datapoints), extracted with STRUCTURE Harvester.

Table S1 Details of the 17 microsatellite loci included in this study.

Table S2 Gill raker counts (GRC) for indigenous and reintroduced North Sea houting (NSH), Baltic houting (BH) and European whitefish (EW) populations across the study area, with supplementary literature values for indigenous NSH populations for which no GRC could be obtained in this study.

Table S3 Estimates of pairwise genetic differentiation (*F*_st_) between source and reintroduced populations of North Sea houting (NSH), Baltic houting (BH) and European whitefish (EW) based on mtDNA data.

Table S4 Tests values for recent demographic expansion or bottlenecks based on mtDNA. Tajima's D, Fu's F, SSD and the raggedness index r with their corresponding *P*-values.

Table S5 amova results based on the microsatellite dataset, with *a priori* taxon affiliation (North Sea houting, Baltic houting, European whitefish) and populations as grouping levels.
